# S100A8-Mediated NLRP3 Inflammasome-Dependent Pyroptosis in Macrophages Facilitates Liver Fibrosis Progression

**DOI:** 10.3390/cells11223579

**Published:** 2022-11-12

**Authors:** Yan Liu, Xuehua Kong, Yan You, Linwei Xiang, Yan Zhang, Rui Wu, Lan Zhou, Liang Duan

**Affiliations:** 1 Key Laboratory of Laboratory Medical Diagnostics, Ministry of Education, Department of Laboratory Medicine, Chongqing Medical University, Chongqing 400016, China; 2 Department of Pathology, The Second Affiliated Hospital of Chongqing Medical University, Chongqing 400010, China; 3 Department of Laboratory Medicine, The First Affiliated Hospital of Chongqing Medical University, Chongqing 400016, China; 4 Department of Laboratory Medicine, The Second Affiliated Hospital of Chongqing Medical University, Chongqing 400010, China

**Keywords:** NLRP3, S100A8, GSDMD, liver fibrosis

## Abstract

NLRP3 inflammasome-dependent pyroptosis has been implicated in liver fibrosis progression. However, the definite intrahepatic cell types that undergo pyroptosis and the underlying mechanism as well as the clinical importance remain unclear. Here, augmented levels of pyroptosis-related indicators GSDMD, IL-1β, and IL-18 were verified in both liver fibrosis patients and CCl4-induced fibrotic mouse model. Confocal imaging of NLRP3 with albumin, F4/80 or α-SMA revealed that enhanced NLRP3 was mainly localized to kupffer cells (KCs), indicating that KCs are major cell types that undergo pyroptosis. Targeting pyroptosis by inhibitor MCC950 attenuated the severity and ameliorated liver function in fibrosis models. In addition, elevated S100A8 in liver fibrosis patients was correlated with pyroptosis-related indicators. S100A8 stimulated pyroptotic death of macrophages, which resulted in activation of human hepatic stellate cell line LX-2 cells and increased collagen deposition. Mechanistically, S100A8 activated TLR4/NF-κB signaling and upregulated its target genes NLRP3, pro-IL-1β, and pro-IL-18 expression, and induced reactive oxygen (ROS) abundance to activate NLRP3 inflammasome, finally leading to pyroptotic cell death in macrophages. More importantly, circulating GSDMD had the optimal predicting value for liver fibrosis progression. In conclusion, S100A8-mediated NLRP3 inflammasome-dependent pyroptosis by TLR4/NF-κB activation and ROS production in macrophages facilitates liver fibrosis progression. The identified GSDMD has the potential to be a biomarker for liver fibrosis evaluation.

## 1. Introduction

Hepatic fibrosis is a wound-healing response characterized by the accumulation of extracellular matrix (ECM) following excessive cell death and chronic liver inflammation due to a variety of etiological factors, including virus infection, alcohol abuse, non-alcoholic steatohepatitis, parasitemia, metabolic disorders, and drugs [1,2]. Early stage liver fibrosis can be stopped or reversed by removing the insults that triggered liver damage and inflammation [3,4]. However, in many cases, liver fibrosis progresses to cirrhosis over time and increases the risk of liver failure and hepatocellular carcinoma [1]. At present, serology examination represents one of the frequently used methods for the diagnosis and evaluation of liver fibrosis, but several limitations still exist, including low sensitivity and specificity, inaccurate disease assessment, and even misdiagnosis. In addition, despite decades of efforts by clinical research, there is no effective therapy for liver fibrosis. Therefore, further elucidating the pathogenesis of liver fibrosis and identifying novel biomarkers that closely reflect disease progression are urgently needed.

NLRP3 (NACHT, LRR, and PYD domains-containing protein 3, cryoporin) inflammasome-dependent pyroptosis, a newly identified inflammatory cell death, participates in multiple diseases, including infection, metabolic disorders, and cancer [5,6]. It starts with the recognition of pathogen-associated molecular patterns (PAMPs) or damage-associated molecular patterns (DAMPs) by extracellular pattern recognition receptors (PRRs) leading to enhanced transcription of NLRP3, pro-IL (interleukin)-1β, and pro-IL-18, continues with the activation of NLRP3 and caspase-1 by multiple intracellular stimulus, and ends with gasdermin D (GSDMD)-mediated formation of membrane pores and the maturation and release of proinflammatory cytokines IL-1β and IL-18 [7]. Due to the proinflammatory property of pyroptosis, the role of uncontrolled pyroptosis caused by aberrant inflammasome activation in inflammation-associated diseases has received considerable attention. Pyroptosis has recently been reported to be associated with pulmonary, renal, and cardiovascular fibrosis [8,9,10]. More importantly, growing evidence suggests a close correlation between NLRP3 inflammasome activation as well as its downstream effectors and liver fibrosis progression. One study reported that hyperactivation of the NLRP3 inflammasome in mice results in hepatocyte pyroptotic death, severe liver inflammation, and fibrosis [11]. Moreover, in a mouse model of non-alcoholic fatty liver disease and non-alcoholic steatohepatitis, NLRP3 inflammasome activation is required for liver inflammation and fibrosis [12,13]. In addition, an in vitro mechanistic study showed that the pyroptosis products IL-1β and IL-18 regulate the activation of hepatic stellate cells (HSCs) and facilitate the development of liver fibrosis [14]. Notably, patients with liver cirrhosis also exhibited elevated levels of circulating GSDMD, IL-1β, and IL-18 in our previous clinical research [15]. Therefore, NLRP3 inflammasome-dependent pyroptosis may serve as a crucial mechanism for the development of liver injury and fibrosis. Nevertheless, the primary occurrence of NLRP3 inflammasome-dependent pyroptosis in which type of cells (hepatocytes, KCs or HSCs), the detailed molecular mechanism regarding how pyroptosis occurs, and its clinical importance during liver fibrosis progression, are still unclear.

S100A8 and S100A9, belonging to the S100 protein family (S100s), are secreted mainly by inflammatory, tumor, and stromal cells exhibiting proinflammatory functions. As two DAMPs, S100A8 and S100A9 have been implicated as inflammation triggers participating in the progression of multiple inflammatory diseases, including rheumatoid arthritis [16], inflammatory bowel [17], and lung disease [18]. Recently, S100A8 and S100A9 were reported to activate NLRP3 inflammasome signaling to promote the pathogenesis of myelodysplastic syndromes [19] and airway obstructive diseases [20]. It is worth noting that our previous study demonstrated an elevated S100A9 in liver fibrosis [21]. Given this, we hypothesized that S100A8 and/or S100A9 may regulate NLRP3 inflammasome-dependent pyroptosis to establish a proinflammatory microenvironment, thereby potentiating the progression of liver fibrosis.

In the present study, the definite cell types that undergo pyroptosis and the underlying mechanism as well as the clinical importance were investigated, aiming to further reveal the pathogenesis of liver fibrosis and identify novel markers and intervention targets. Here, we observed that the macrophage was the major cell type that underwent NLRP3 inflammasome-dependent pyroptosis in liver fibrosis, which could be mediated by S100A8-induced Toll-like receptor 4 (TLR4)/NF-κB activation and ROS generation. Furthermore, inhibiting NLRP3 inflammasome-dependent pyroptosis effectively attenuated liver injury and fibrosis severity in a carbon tetrachloride (CCl4)-induced liver fibrosis mouse model. More importantly, the pyroptosis-related indicator GSDMD had a high predictive value for the onset and progression of liver fibrosis.

## 2. Materials and Methods

### 2.1. Human Samples

A total of eighty-nine patients with liver fibrosis were enrolled in the current study between March 2020 and December 2021 at the Second Affiliated Hospital of Chongqing Medical University. Diagnosis was primarily established by histology as well as other methods, such as serological, imaging examination, and medical history. Etiologies, such as viral infection, alcohol consumption, and autoimmunity were determined according to serological and histological findings. The sections for liver histology were examined independently by two experienced pathologists who were unaware of the clinical status. Liver fibrosis grading was assessed according to Batts-Ludwing scores (Fibrosis F0 to F4) [22]. Additionally, sixty age- and gender-matched healthy volunteers who did not have evidence of liver diseases or other chronic disorders were enrolled as healthy controls (HCs). Moreover, five normal liver samples were obtained from healthy controls who underwent liver biopsy to exclude malignancy. The peripheral blood was centrifuged for 10 min to obtain serum. Then, the serum was stored at −80 °C for further examination. This study protocol was in accordance with the ethical guidelines of the Declaration of Helsinki Principles. Informed written consent was obtained from all patients and the study was approved by the Institutional Ethics Committee at the Second Hospital affiliated with Chongqing Medical University (No. 2020-65). Patient characteristics are summarized in Table 1.

### 2.2. CCl4-Induced Liver Fibrosis Mouse Models

Herein, 6 to 8-week-old male C57BL/6 mice were randomly grouped. For toxic liver fibrosis, they were given intraperitoneal (i.p.) injections of CCl4 (2.5 mL/kg body weight, dissolved in olive oil at a ratio of 1:5) or vehicle (olive oil) (O108686, Aladdin, Fengxian, Shanghai, China) two times per week for 4, 6 or 8 weeks (*n* = 5/group). The mice were sacrificed at 72 h after the final CCl4 injection.

To assess the role of NLRP3 signaling in the mouse model of liver fibrosis, 6 to 8-week-old male mice were randomly divided into three groups. The CCl4 group were given intraperitoneal (i.p.) injections of CCl4 (2.5 mL/kg body weight, dissolved in olive oil at a ratio of 1:5). The (CCl4+MCC950) group were injected (i.p.) with MCC950 (10 mg/kg body weight in 0.9% NaCl) (CP-456773, Selleck, Houston, TX, USA) every second day at the same time as the CCl4 injection up to 8 weeks, while the control group (CCl4+saline) was administrated a comparable volume of 0.9% NaCl (*n* = 5/group). The mice were sacrificed at 72 h after the final CCl4 injection. All animal experiments were approved and conducted in accordance with the guidelines established by the Hospital Animal Care and Use Committee for Laboratory Animal Research in the Second Affiliated Hospital of Chongqing Medical University (No. 2020-65).

### 2.3. Mouse Serum and Liver Samples Preparation

At the end of the treatment, all mice were anesthetized and the blood samples were taken via cardiac puncture. The mouse blood was centrifuged at 3500× *g* rpm at 4 °C for 15 min and then for 10 min to remove any remaining cellular debris. Finally, the serum was stored at −80 °C for further examination. Then, the liver was harvested. A representative section was fixed in 4% paraformaldehyde for 24 h and embedded in paraffin, and the rest of the liver tissue was stored in liquid nitrogen.

### 2.4. Analysis of Liver Function, Liver Pathology, and Fibrosis

The serum alanine aminotransferase (ALT), aspartate aminotransferase (AST), and total proteins (TP) were assayed by the Autoanalyzer Hitachi 7600-110. H&E staining was used to assess the pathological morphology of the liver. Sirius Red staining was used to demonstrate collagen deposition. The stained sections were observed and photographed under a light microscope (Nikon E400, Chiyoda, Tokyo, Japan).

### 2.5. Immunohistochemical Staining

The formalin-fixed, paraffin-embedded human and mouse liver tissue sections were subjected to IHC staining. Briefly, the sections were deparaffinized, hydrated, and subjected to antigen retrieval by incubating the slides in a pressure cooker for 15 min in 0.01 M citrate buffer and then incubated with 0.3% hydrogen peroxide (H_2_O_2_) in methanol for 10 min to block endogenous peroxidase activity. Then, the sections were incubated with primary antibodies against α-smooth muscle actin (α-SMA) (14395-1-AP, Proteintech, Wuhan, Hubei, China), NLRP3 (19771-1-AP, Proteintech, Wuhan, Hubei, China), GSDMD (20770-1-AP, Proteintech, Wuhan, Hubei, China), IL-1β (16806-1-AP, Proteintech, Wuhan, Hubei, China), S100A8 (ab92331, Abcam, Cambridge, England, UK) or S100A9 (ab63818, Abcam, Cambridge, England, UK) overnight at 4 °C. The cells were washed with PBS and stained with anti-rabbit IHC Secondary Antibody Kit (SP-9001, Zhongshan Golden Bridge, Haidian, Beijing, China). Finally, the sections were visualized with 0.05% 3,3-diamino-benzidine tetrachloride (DAB) until the desired brown reaction product was obtained. The stained sections were observed and photographed under a light microscope (Nikon E400, Chiyoda, Tokyo, Japan).

### 2.6. Immunofluorescence Staining

The formalin-fixed, paraffin-embedded human and mouse liver tissue sections were processed for immunofluorescence staining. In brief, the liver sections were deparaffinized, hydrated, subjected to antigen retrieval, permeabilization, and serum blocking, and then incubated with primary antibody overnight at 4 °C for double immunofluorescence staining. The primary antibodies used were as follows: Rabbit anti-NLRP3 (19771-1-AP, Proteintech, Wuhan, Hubei, China) with rat anti-albumin (MAB1455-SP, R&D, Minneapolis, MN, USA) or with mouse anti-F4/80 (14-4801-85, Invitrogen, Carlsbad, CA, USA), rat anti-NLRP3 (MAB7578-SP, R&D, Minneapolis, Minnesota, USA) with rabbit anti-α-SMA (14395-1-AP, Proteintech, Wuhan, Hubei, China). Then, the sections were washed three times with PBS. Alexa Fluor 647-conjugated goat anti-rabbit secondary antibody (bs-0295G-AF647, Bioss, Tongzhou, Beijing, China) and Alexa Fluor 488-conjugated goat anti-mouse secondary antibody (bs-0296G-AF488, Bioss, Tongzhou, Beijing, China) or goat anti-rat secondary antibody (bs-0293G-AF488, Bioss, Tongzhou, Beijing, China), Alexa Fluor 647-conjugated goat anti-rat secondary antibody (bs-0293G-AF647, Bioss, Tongzhou, Beijing, China) and Alexa Fluor 488-conjugated goat anti-rabbit secondary antibody (bs-0295G-AF488, Bioss, Tongzhou, Beijing, China) were used for 1 h at room temperature in the dark. Then, the sections were washed with PBS three times, and nuclei were stained with DAPI for 5 min. The sections were washed with PBS three times and mounted with antifade mounting medium (Beyotime, Songjiang, Shanghai, China). Finally, the sections were observed under a confocal microscope (Lecia, Weztlar, Germany).

### 2.7. Enzyme-Linked Immunosorbent Assay (ELISA)

Protein of interest in serum or cell supernatant was detected by ELISA according to the manufacturer’s instructions. Detailed ELISA kits were as follows: Mouse S100A8 (E-EL-M3048, elabscience, Wuhan, Hubei, China), mouse IL-1β (VAL601, Novus, Littleton, CO, USA), mouse IL-18 (E-EL-M0730c, elabscience, Wuhan, Hubei, China), mouse GSDMD (JL46371-96T, JiangLai, Baoshan, Shanghai, China), human S100A8 (E-EL-H1289c, elabscience, Wuhan, Hubei, China), human IL-1β (Mengbio, Shapingba, Chongqing, China), human IL-18 (Mengbio, Shapingba, Chongqing, China), and human GSDMD (Mengbio, Shapingba, Chongqing, China).

### 2.8. Preparation of Recombinant Proteins

The pGST-moluc and pGST-moluc-S100A8 have been described previously [23]. Briefly, the two plasmids were cloned into E. coil (BL21) by calcium chloride transformation. Then, 0.1 mM isopropylthio-β-D-galactoside was used to induce the expression of GST and GST-hS100A8 protein for 8 h at 14 °C. After incubation, the bacteria were centrifuged at 5000× *g* for 10 min and the pellet was resuspended in PBS supplemented with protease inhibitor and 0.1% Triton X-100 and lysed by sonication. Then, the supernatant was collected and incubated with glutathione-sepharose 4B beads (Amersham Biosciences) for 3 h at 4 °C. Recombinant GST-hS100A8 (rhS100A8) or GST bound to the beads was eluted by an elution buffer with reduced glutathione on ice. Finally, the recombinant rhS100A8 or control GST protein was filtered with a 0.22 µm membrane and stored at −80 °C.

### 2.9. Cell Culture and In Vitro Treatment

Human monocyte THP-1 cells were cultured in 5% CO_2_ at 37 °C in 1640-RPMI medium supplemented with 10% fetal bovine serum (FBS, HyClone, Logan, UT, USA), 100 U/mL penicillin, and 100 µg/mL streptomycin (HyClone). THP-1 cells were stimulated with PMA (50 µg/mL) (Sigma, Saint Louis, MO, USA) for 4 h to differentiate them into macrophages, then washed two times with PBS and maintained in fresh medium for further experiments.

To induce pyroptosis in THP-1 differentiated macrophages, high dose of lipopolysaccharide (LPS) (1 µg/mL, L2630, Sigma-Aldrich, Saint Louis, MO, USA) was added to the culture media. In certain experiments, THP-1 differentiated macrophages were stimulated with 2, 5 or 10 µg/mL rhS100A8 for 24 h to extract RNA or for 48 h to extract protein. To explore the role of NF-κB signaling and ROS production in S100A8-induced pyroptosis, THP-1 differentiated macrophages were treated with the NF-κB inhibitor BAY 11-7082 (10 µM, Beyotime, Songjiang, Shanghai, China) or NADPH oxidase (NOX) inhibitor diphenylene iodonium (DPI, 10 µM, S8639, Selleck, Houston, Texas, USA) for 1 h prior to rhS100A8 stimulation (5 µg/mL). To investigate the endogenous PRR of S100A8, THP-1 differentiated macrophages were pretreated with the TLR4 inhibitor TAK-242 (10 µM, S7455, Selleck, Houston, Texas, USA) or the receptor for advanced end products (RAGE) inhibitor FPS-ZM1 (10 µM, S8185, Selleck, Houston, Texas, USA) for 1 h and then stimulated with rhS100A8 (5 µg/mL).

The human hepatic stellate cell line LX-2 was maintained in 5% CO_2_ at 37 °C in Dulbecco’s modified Eagle medium (DMEM, Gibco, Grand Island, NY, USA) with 10% fetal bovine serum (FBS, HyClone, Logan, UT, USA), 100 U/mL penicillin, and 100 µg/mL streptomycin (HyClone, Logan, UT, USA). LX-2 cells were exposed to conditioned media (CM) from rhS100A8-treated THP-1 macrophages and an equal volume of new DMEM medium for 24 h to extract RNA or for 48 h to extract protein. To confirm that macrophage pyroptosis triggers the activation of HSCs, LX-2 cells were treated with the conditioned medium from THP-1 macrophages exposed to 5 µ/mL of rhS100A8 with or without 1 h of MCC950 pretreatment (1 µM).

### 2.10. RNA Extraction and Quantitative Real-Time PCR

Total cellular RNA was isolated using Trizol (Invitrogen, Carlsbad, CA, USA) according to the manufacturer’s instructions. Briefly, 1 µg of total RNA was reverse-transcribed to cDNA via an Evo M-MLV RT mix kit with gDNA clean (AG11728, Accurate Biotechnology, Changsha, Hunan, China) according to the manufacturer’s protocol. The mRNA levels of NLRP3, pro-IL-1β, pro-IL-18, collagen I (COLIA1), α-SMA, and transforming growth factor-β (TGF-β) were analyzed with the CFX96 real-time PCR detection system (Bio-Rad, Richmond, CA, USA) using SYBR Green dye (Biomake, Houston, TX, USA). Primer sequences are summarized in Table 2. GAPDH was used as a reference control. The fold changes in gene expression were calculated by the 2-ΔΔCT method.

### 2.11. Western Blot

Treated cells were collected and lysed in RIPA lysis buffer containing phosphatase and protease inhibitors. The BCA protein assay (abs9232, Absin, Pudong New District, Shanghai, China) was used to assess the protein concentrations. Samples containing equal amounts (30 µg) of proteins were separated by 10% SDS-PAGE and then transferred to polyvinylidene fluoride membranes. Then, the membranes were blocked with 5% bovine serum albumin and incubated overnight at 4 °C with primary antibody against NLRP3 (19771-1-AP, Proteintech, Wuhan, Hubei, China), GSDMD (20770-1-AP, Proteintech, Wuhan, Hubei, China), IL-1β (16806-1-AP, Proteintech, Wuhan, Hubei, China), cleaved caspase-1 (4199T, Cell Signaling Technology, Boston, MA, USA), α-SMA (14395-1-AP, Proteintech, Wuhan, Hubei, China), COL1A1 (66761-1-lg, Proteintech, Wuhan, Hubei, China), TGF-β (MAB1835-SP, R&D, Minneapolis, Minnesota, USA), total NF-κB p65 (10745-1-AP, Proteintech, Wuhan, Hubei, China), phospho-NF-κB p65 (p-p65) (3033, Cell Signaling Technology, Boston, MA, USA), total IKKα (db2315, diagbio, Hangzhou, Zhejiang, China), phospho-IKKα (p-IKKα) (2697, Cell Signaling Technology, Boston, MA, USA), and β-actin (Zoonbio Biotechnology, Nanjing, Jiangsu, China). The next day, after incubation with goat-anti-rabbit or goat-anti-mouse secondary antibodies, the samples were conjugated with horseradish peroxidase for 1 h at 37 °C, and the immune complexes were detected by enhanced chemiluminescence (ECL, Millipore, Boston, MA, USA).

### 2.12. Flow Cytometry

The production of ROS in THP-1 macrophages was measured using the dichlorodihydrofluorescein diacetate (H2DCFDA) probe (S9687, Selleck, USA) according to the manufacturer’s recommendation. In brief, THP-1 macrophages were stimulated with rhS100A8 (5 µg/mL) or the control protein GST (5 µg/mL) for 6 h. THP-1 macrophages were collected and washed three times with serum-free medium, and subsequently incubated in serum-free medium containing 10 µM H2DCFDA probe at 37 °C for 30 min in the dark. Then, the media containing H2DCFDA was removed and washed two times with PBS, and the fluorescence intensity of the cells was analyzed by flow cytometry (CytoFLEX). Cells that had been incubated without H2DCFDA were used as negative controls. To detect pyroptotic death in THP-1 macrophages that had been treated with rhS100A8 or control protein GST for 24 h, FLICA 660-YVAD-FMK (FLICA 660 in vitro Active Caspase-1 Detection Kit; ImmunoChemistry Technologies, Davis, CA, USA) was used according to the manufacturer’s instructions and propidium iodide (PI) was used to mark cells with membrane pores (Life Technologies, Carlsbad, CA, USA). Flow cytometry measurements were performed three times for each treatment. The mean fluorescence intensity was quantified usingFlowJo v10.8.1 (FlowJo LLC, Ashland, OR, USA).

### 2.13. Statistical Analysis

All data were analyzed using SPSS 17.0 (IBM Corp., Armonk, NY, USA). Human data were not normally distributed continuous variables and were expressed as the median and interquartile range (IQR). Animal data were expressed as the mean ± standard deviation (SD). Statistical analysis of serum levels of GSDMD, IL-1β, and IL-18 in liver fibrosis patients was determined by the Kruskal-Wallis or Mann-Whitney test. Correlation coefficients (r) were calculated using Spearman correlation. ROC curves were generated to classify patients into different groups, as well as to evaluate the predictive power of serum GSDMD, IL-1β, and IL-18 levels via the calculation of AUC. Differences between multiple groups in the in vitro cell experiments were evaluated using a *t*-test or one-way analysis of variance. A *p*-value < 0.05 was considered statistically significant.

## 3. Results

### 3.1. NLRP3 Inflammasome-Dependent Pyroptosis Occurs in Liver Fibrosis

**Figure 1:**
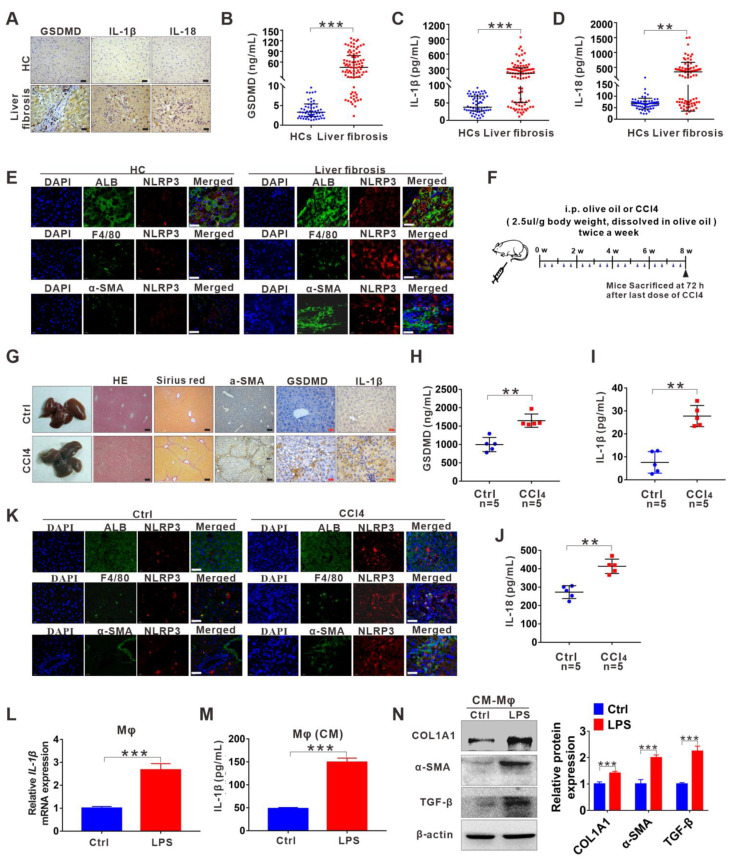
NLRP3 inflammasome-dependent pyroptosis occurs in liver fibrosis. (**A**) IHC staining for GSDMD, IL-1β, and IL-18 in liver sections from liver fibrosis patients and HCs. Scale bar: 40 µm. (**B**–**D**) ELISA analyses of serum levels of GSDMD (**B**), IL-1β (**C**), and IL-18 (**D**) in liver fibrosis patients (*n* = 89) and HCs (*n* = 60). (**E**) Representative immunofluorescence images of NLRP3 (red) and albumin (hepatocyte marker) (top), F4/80 (KC marker) (middle) or α-SMA (HSC marker) (bottom) (green) from the human fibrotic liver tissues. Scale bar: 40 µm. (**F**) Schematic diagram of the study. Liver fibrosis was induced by CCl4 injection for 8 weeks. (**G**) Representative mouse liver histology of H&E, Sirius Red staining, and IHC staining for α-SMA, GSDMD, and IL-1β. Black scale bar: 100 µm; Red scale bar: 50 µm. (**H**–**J**) ELISA analyses for serum levels of GSDMD (H), IL-1β (I), and IL-18 (J) in CCl4 group mouse (*n* = 5) and vehicle group mouse (*n* = 5). (**K**) Representative immunofluorescence images of NLRP3 (red) and albumin (hepatocyte marker) (top), F4/80 (KC marker) (middle) or α-SMA (HSC marker) (bottom) (green) from the 8-week CCl4-treated mouse liver. The vehicle group mouse liver was used as a control. Scale bar: 40 µm. (**L**) The qRT-PCR analysis for mRNA levels of IL-1β in THP-1 macrophages treated with LPS to induce pyroptosis. (**M**) ELISA analysis for IL-1β expression in supernatants from THP-1. (**N**) Western blot analysis of COL1A1, α-SMA, and TGF-β expression in LX-2 cells which were exposed to CM from LPS-treated THP-1 macrophages. The protein expression was quantified by densitometry and normalized to β-actin and are shown as fold changes relative to the control group (right panel). ** *p* < 0.01, *** *p* < 0.001.

Herein, we examined the pyroptosis-related indicators NLRP3, GSDMD, IL-18, and IL-1β. Immunohistochemical (IHC) analysis revealed that the expression of hepatic GSDMD, IL-1β, and IL-18 was significantly upregulated in patients with liver fibrosis compared to HCs (Figure 1A and Figure S1A). Moreover, serological data of GSDMD, IL-1β, and IL-18 supported this IHC result (Figure 1B–D). To further investigate in which types of cells (hepatocytes, KCs, or HSCs) NLRP3 inflammasome-dependent pyroptosis mainly occurs during the process of liver fibrosis, we examined the co-location of NLRP3 with the hepatocyte marker albumin, the KC marker F4/80 or the HSC marker α-smooth muscle actin (α-SMA) in human fibrotic liver tissues. We observed the enhanced expression of NLRP3 in patients with liver fibrosis compared to HCs and NLRP3 was mainly localized to hepatocytes and KCs but not HSCs, especially KCs, indicating that KCs are major cell types that undergo pyroptosis (Figure 1E). Then, we further validated the above results with a CCl4-induced liver fibrosis mouse model (Figure 1F). H&E, Sirius Red, and α-SMA staining proved that we successfully established a mouse model of liver fibrosis (Figure 1G and Figure S1B). Moreover, consistent with the human data, GSDMD and IL-1β expression were significantly increased in the liver from the liver fibrosis mouse model compared with the control (Figure 1G and Figure S1B). Furthermore, serum levels of GSDMD, IL-18, and IL-1β were markedly enhanced in mouse models of liver fibrosis (Figure 1H–J). As expected, double immunolabelling in mouse liver sections also indicated that the activation of NLRP3 occurred mainly in KCs (Figure 1K). To investigate the effects of macrophage pyroptosis on the activation of HSCs and liver fibrosis in vitro, we induced pyroptotic death in THP-1 macrophages using the pyroptosis inducer lipopolysaccharide (LPS) and collected the CM to treat LX-2 cells. The protein levels of IL-1β in the cells and supernatants were confirmed (Figure 1M). The protein levels of HSC activation and the collagen deposition markers COLIA1, α-SMA, and TGF-β were significantly higher in LPS-CM-cultured LX-2 cells than in the control group (Figure 1N).

### 3.2. Inhibition of NLRP3 Inflammasome-Dependent Pyroptosis Alleviates Liver Fibrosis Progression

**Figure 2:**
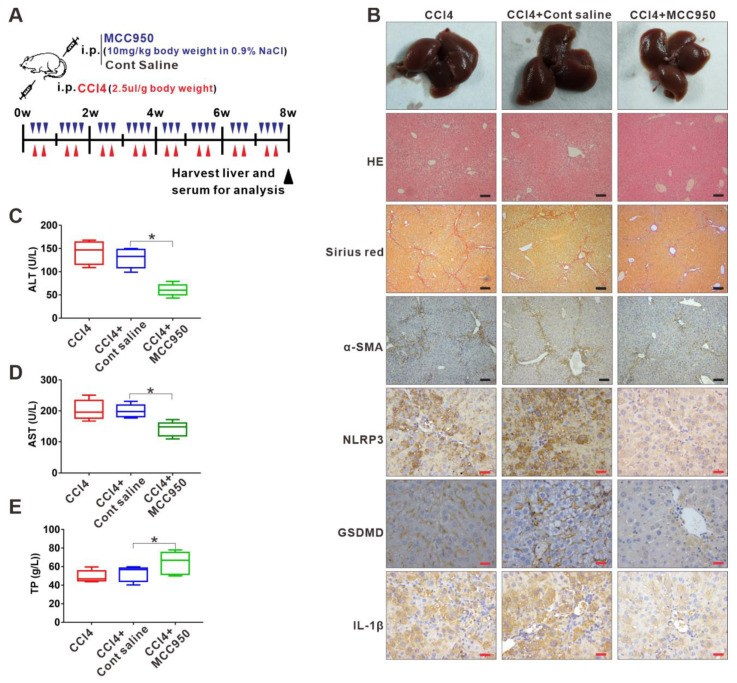
Inhibition of NLRP3 inflammasome-dependent pyroptosis alleviates liver fibrosis progression. (**A**) Experimental protocol of NLRP3 inhibitor MCC950 or saline application based on CCl4 injection in mice. (**B**) Representative liver histology of H&E and Sirius Red staining. The expression of α-SMA, NLRP3, GSDMD, and IL-1β was determined by immunohistochemistry. Black scale bar: 100 µm; Red scale bar: 50 µm. (**C**–**E**) Serum levels of ALT, AST, and TP were measured. * *p* < 0.05.

Given that NLRP3 inflammasome-dependent pyroptosis was involved in the liver fibrosis, we used a specific molecular inhibitor of NLRP3 (MCC950) to treat the liver fibrosis mouse model (Figure 2A), aiming to explore whether targeting the NLRP3 inflammasome-dependent pyroptosis can attenuate liver fibrosis progression. IHC analysis demonstrated that the MCC950 treatment significantly decreased the expression of pyroptosis-related indicators NLRP3, GSDMD, and IL-1β in the fibrotic livers (Figure 2B and Figure S1C). Importantly, the MCC950 treatment reduced liver injury and fibrosis severity, as analyzed by histology, collagen, and α-SMA via HE, Sirius Red, and IHC staining, respectively (Figure 2B and Figure S1C). Serum ALT and AST levels in the serum were notably lower in the MCC950-treated group than in the saline-treated group, while the serum levels of total proteins (TP) were significantly increased in the MCC950-treated group compared to the saline-treated group, indicating an improvement in liver function after MCC950 treatment (Figure 2C–E).

### 3.3. DAMP S100A8 along with NLRP3 Inflammasome-Dependent Pyroptosis Is Positively Related to the Progression of Liver Fibrosis

**Figure 3:**
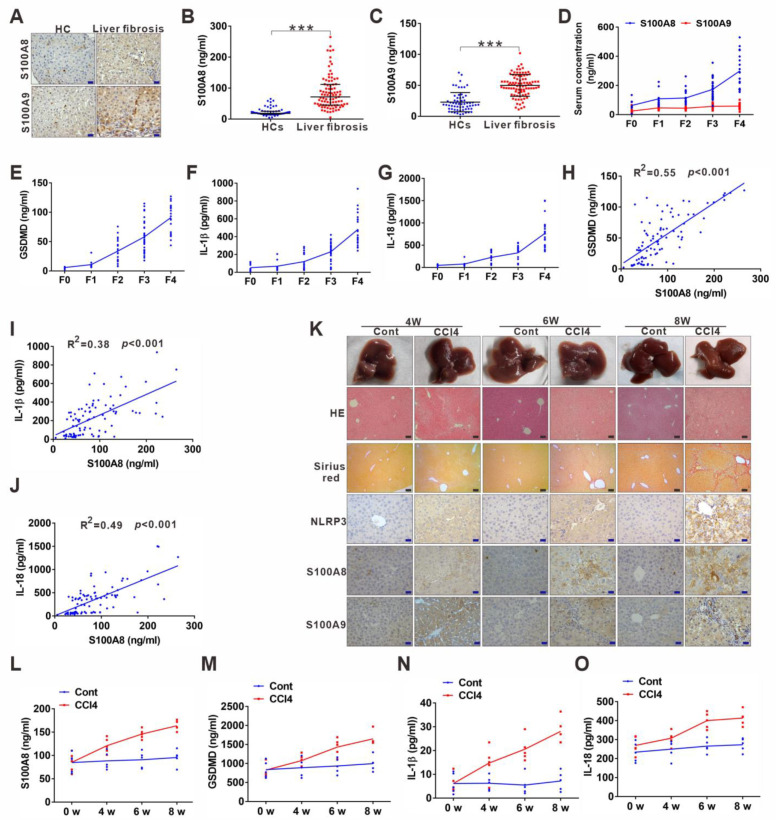
DAMP S100A8 along with NLRP3 inflammasome-dependent pyroptosis is positively related to the progression of liver fibrosis. (**A**) Representative IHC images for S100A8 and S100A9 in liver sections from liver fibrosis patients and HCs. (**B**,**C**) ELISA analyses for serum levels of S100A8 and S100A9 in liver fibrosis patients and HCs. (**D**) Comparison of serum S100A8 and S100A9 levels in liver fibrosis patients with different phases. (**E**–**G**) Distribution of serum GSDMD (**E**), IL-1β (**F**), and IL-18 (**G**) levels in liver fibrosis patients with different phases (F0–4). (**H**–**J**) Correlation between serum S100A8 levels and GSDMD (**H**), IL-1β (**I**) or IL-18 (**J**) levels in liver fibrosis patients. (**K**) Representative mouse liver morphology and staining with H&E and Sirius Red. (**L**–**O**) IHC staining of mouse liver sections for NLRP3, S100A8, and S100A9. Black scale bar: 100 µm; Red scale bar: 50 µm. ELISA analyses for serum levels of S100A8 (**L**), GSDMD (**M**), IL-1β (*n*), and IL-18 (**O**) in 4-, 6-, and 8 week-mouse models of liver fibrosis. *** *p* < 0.001.

Hepatic inflammation is the main initiator of liver injury and fibrosis. As two members of DAMPs, S100A8 and S100A9, can serve as triggering factors and amplifiers of inflammation [24], and we have previously found that S100A9 increases in liver fibrosis [21]. Therefore, we further addressed their relationship with hepatic inflammation and fibrosis. IHC and enzyme-linked immunosorbent assay (ELISA) results showed that S100A8 and S100A9 were both significantly elevated in liver fibrosis patients compared to HCs (Figure 3A–C and Figure S1D). Notably, S100A8 increased more dramatically than S100A9 during the progression of liver fibrosis from fibrosis F0 to F4 (Figure 3D). Additionally, the levels of the pyroptosis-related indicators GSDMD, IL-18, and IL-1β were consistent with those of S100A8, exhibiting a gradual elevation from F0 to F4 (3E–G). Moreover, S100A8 levels were found to be positively correlated with the pyroptosis-related indicators GSDMD, IL-18, and IL-1β levels in LF patients (Figure 3H–J). Then, we conducted CCl4-induced mouse liver fibrosis models (4/6/8 weeks) to further verify the results mentioned above. The progression of liver fibrosis was proven by H&E, Sirius Red, and α-SMA staining from 4 to 8 weeks (Figure 3K). Staining signals of the pyroptosis mediator NLRP3 alone with S100A8 were gradually increased with the aggravation of liver fibrosis in mouse models from 4 to 8 weeks (Figure 3K and Figure S1E,F). Similar to the human data, the increase in S100A9 was not dramatic during the progression (Figure 3K and Figure S1G). Furthermore, serum S100A8 and pyroptosis-related indicators GSDMD, IL-1β, and IL-18 levels were all gradually augmented during the progression of the liver fibrosis model (Figure 3L–O).

### 3.4. S100A8-Mediated NLRP3 Inflammasome-Dependent Pyroptotic Macrophage Death Amplifies the Activation of Human Hepatic Stellate Cells

**Figure 4:**
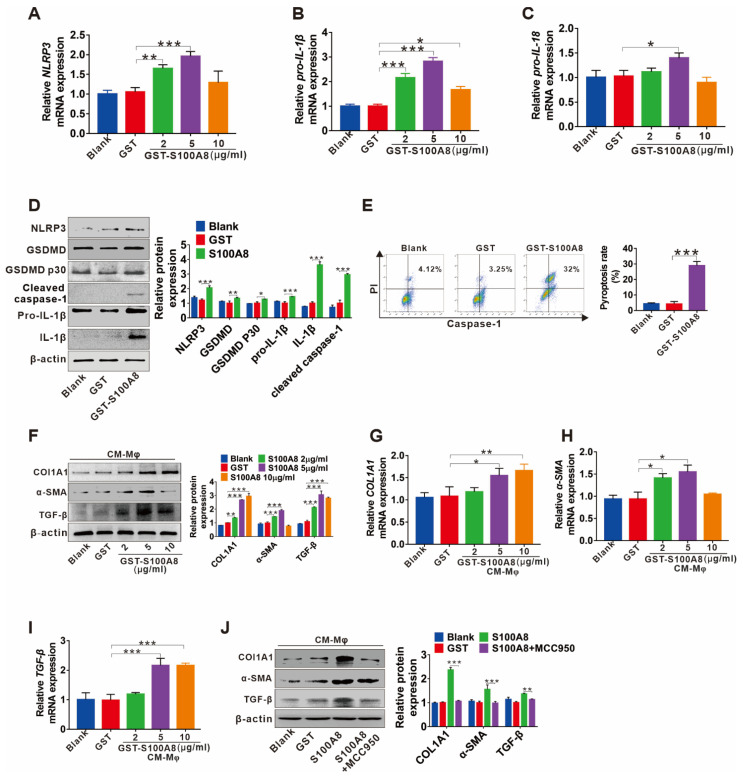
S100A8-mediated NLRP3 inflammasome-dependent pyroptotic macrophage death amplify the activation of human hepatic stellate cells. (**A**–**C**) The qRT–PCR analysis for the mRNA levels of NLRP3, pro-IL-1β, and pro-IL-18 in THP-1 macrophages treated with 0, 2, 5 or 10 µg/mL rhS100A8 or 5 µg/mL GST for 24 h. (**D**) The protein levels of NLRP3, GSDMD, GSDMD P30, pro-IL-1β, mature IL-1β, and cleaved caspase-1 were detected by Western blot in THP-1 macrophages treated with 5 µg/mL GST or rhS100A8. The protein expression was quantified by densitometry and normalized to β-actin and are shown as fold changes relative to the GST group (right panel). (**E**) PI and active caspase-1 double staining of pyroptotic cell death by flow cytometry in THP-1 macrophages treated with 5 µg/mL GST or rhS100A8. (**F**–**I**) Western blot analysis (**F**) and qRT-PCR analysis (**G–I**) of COL1A1, α-SMA, and TGF-β in LX-2 cells exposed to CM from THP-1 macrophages that were treated with 0, 2, 5 or 10 µg/mL of rhS100A8 or 5 µg/mL GST. (**J**) Western blot analysis of COL1A1, α-SMA, and TGF-β in LX-2 cells exposed to CM from THP-1 macrophages that were treated with 5 µg/mL of rhS100A8 with or without 1 h of MCC950 pretreatment. The protein expression was quantified by densitometry and normalized to β-actin and are shown as fold changes relative to the GST group (right panel). * *p* < 0.05, ** *p* < 0.01, *** *p* < 0.001.

DAMPs can activate the NLRP3 inflammasome and trigger persistent inflammation, contributing to fibrogenesis of the kidney [25] and lung [26]. Here, we further explored whether S100A8 can promote liver fibrosis by inducing NLRP3 inflammasome-dependent pyroptotic death in macrophages. The mRNA levels of NLRP3, pro-IL-1β, and pro-IL-18 for priming the NLRP3 inflammasome were upregulated by various concentrations of recombinant human GST-hS100A8 (rhS100A8) protein (2, 5, 10 µg/mL) treatment (Figure 4A–C). In addition, rhS100A8 (5 µg/mL) markedly increased the expression of proteins downstream of NLRP3 inflammasome activation, including cleaved GSDMD (GSDMD p30), cleaved caspase-1, and bioactive IL-1β in THP-1 macrophages (Figure 4D), as well as elevated IL-1β levels in supernatants (Figure S2). Moreover, the rhS100A8 treatment resulted in a significant increase in the number of proptotic THP-1 macrophages detected by caspase-1/PI double staining using FCM (Figure 4E). These data suggested that S100A8 could induce the activation of NLRP3 inflammasome signaling and finally lead to pyroptotic cell death in THP-1 macrophages. Furthermore, to determine whether pyroptoic cell death in macrophages induced by S100A8 was involved in HSC hyperactivation, LX-2 cells were treated with CM from rhS100A8-stimulated THP-1 macrophages, and the fibrotic markers TGF-β, COLIA1, and α-SMA were analyzed. The mRNA and protein levels of the COL1A1, α-SMA, and TGF-β in LX-2 cells were elevated by CM from various concentrations of rhS100A8-treated THP-1 macrophages (Figure 4F–I), which could be blocked by the NLRP3 inhibitor MCC950 (Figure 4J).

### 3.5. TLR4/NF-κB Signaling Cascade and ROS Abundance Are Responsible for S100A8-Induced NLRP3 Inflammasome-Dependent Pyroptotic Death in Macrophages

**Figure 5:**
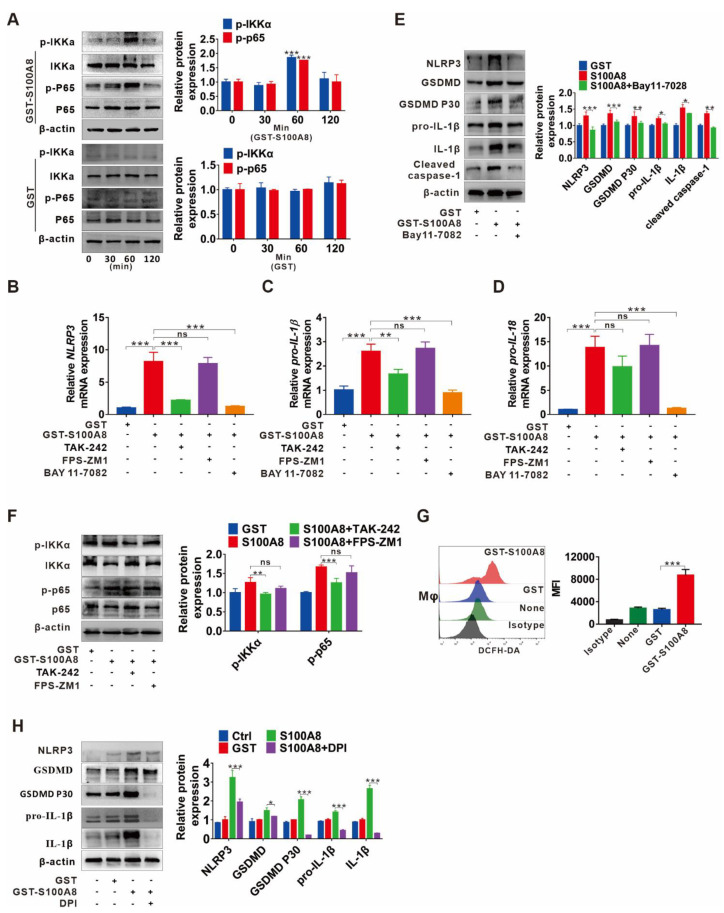
TLR4/NF-κB signaling cascade and ROS abundance are responsible for S100A8-induced NLRP3 inflammasome-dependent pyroptotic death in macrophages. (**A**) Western blot analysis of p65, p-p65, IKKα, and p-IKKα expression in THP-1 macrophages treated with GST-rhS100A8 or GST for 0, 30, 60 or 120 min. The protein expression was quantified by densitometry and normalized to β-actin and are shown as fold changes relative to the 0 min group (right panel). (**B**–**E**) THP-1 macrophages were exposed to 5 µg/mL rhS100A8 with or without 1 h of BAY 11-7082, TAK-242 or FPS-ZM1 pretreatment. The qRT-PCR analysis was performed to detect the mRNA levels of NLRP3 (**B**), pro-IL-1β (**C**), and pro-IL-18 (**D**). Western blot analysis was used to determine the protein expression of NLRP3, GSDMD, GSDMD P30, pro-IL-1β, mature IL-1β, and cleaved caspase-1 (**E**). The protein expression was quantified by densitometry and normalized to β-actin and are shown as fold changes relative to the GST group (right panel). (**F**) THP-1 macrophages were pretreated with TAK-242 or FPS-ZM1 for 1 h and then exposed to 5 µg/mL of rhS100A8. Western blot analysis was used to determine the expression of p-p65 and p-IKKα. The protein expression was quantified by densitometry and normalized to β-actin and are shown as fold changes relative to the GST group (right panel). (**G**) Flow cytometry analysis of ROS levels in THP-1 macrophages treated with rhS100A8 for 6 h. (**H**) THP-1 macrophages were exposed to 5 µg/mL of rhS100A8 with or without 1 h of DPI pretreatment. Protein expression levels of NLRP3, GSDMD, GSDMD P30, pro-IL-1β, and mature IL-1β were determined by Western blot. The protein expression was quantified by densitometry and normalized to β-actin and are shown as fold changes relative to the GST group (right panel); ns, not significant; * *p* < 0.05, ** *p* < 0.01, *** *p* < 0.001.

Since we have observed that S100A8 could induce pyroptotic death in macrophages, we then investigated the potential molecular mechanism. First, we focused on NF-κB activation, a crucial mediator of the priming step for NLRP3 inflammasome-mediated pyroptosis. The protein levels of p-IKKα and p-p65 were enhanced in response to GST-rhS100A8 but not the control GST treatment within 60 min (Figure 5A). In addition, treatment with the NF-κB inhibitor BAY 11-7082 notably reversed S100A8-induced upregulation of the mRNA levels of NLRP3, pro-IL-1β, and pro-IL-18 (Figure 5B–D). Moreover, a similar tendency was confirmed by analysis for protein levels of pyroptosis-related indicators, including NLRP3, GSDMD, GSDMD P30, pro-IL-1β, IL-1β, and cleaved caspase-1 (Figure 5E), suggesting that activation of NF-κB participates in S100A8-induced pyroptosis. It is known that S100A8 is an endogenous ligand of PRRs, including TLR4 [24] and RAGE [27]. Then, we searched whether TLR4 or RAGE transduces S100A8-induced activation of NF-κB signaling as well as the NLRP3 inflammasome. Increased levels of p-p65 and p-IKKα stimulated by S100A8 were partially inhibited by the TLR4 inhibitor TAK-242, while the RAGE inhibitor FPS-ZM1 had fewer effects (Figure 5F). Similarly, inhibition of TLR4 by TAK-242 markedly reduced the mRNA expression of NLRP3, pro-IL-1β, and pro-IL-18, while inhibition of RAGE had minor effects (Figure 5B–D).

As a direct trigger and amplifier of NLRP3 inflammasome activation, ROS is reported to be closely associated with liver fibrosis progression [28]. In peripheral blood of mononuclear cells and HaCaT keratinocytes, ROS production can be induced by S100A8 via increasing NADPH oxidase (NOX) activity [29,30]. Then, we investigated whether S100A8 can directly induce ROS production and mediate NLRP3 inflammasome-dependent pyroptosis. Here, the augmentation of overall ROS levels in THP-1 macrophages was detected after stimulation with S100A8 by DCFH-DA fluorescent probe analysis (Figure 5G). In contrast, suppressing ROS production with the inhibitor DPI clearly attenuated the S100A8-mediated expression of pyroptosis-related indicators NLRP3, GSDMD, pro-IL-1β, mature IL-1β, and GSDMD p30 (Figure 5H), suggesting the important role of ROS in S100A8-mediated NLRP3 inflammasome-dependent pyroptosis.

### 3.6. The Potential Predictive Powers of S100A8, GSDMD, IL-1β, and IL-18 for the Occurrence and Severity of Liver Fibrosis

**Figure 6:**
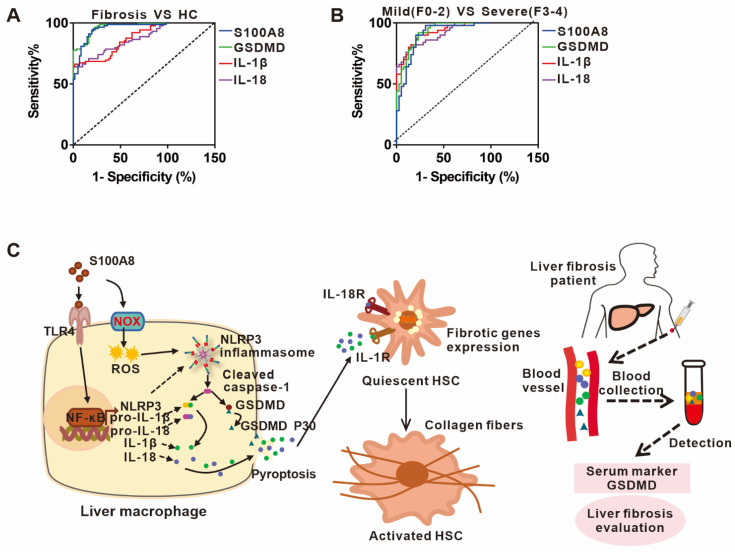
The potential predictive powers of S100A8, GSDMD, IL-1β, and IL-18 for the occurrence and severity of liver fibrosis. (**A**) ROC curves of serum S100A8, GSDMD, IL-1β, and IL-18 for distinguishing liver fibrosis patients from HCs. (**B**) ROC curve, of serum S100A8, GSDMD, IL-1β, and IL-18 for detecting moderate-to-severe liver fibrosis from no or mild liver fibrosis in liver fibrosis patients. (**C**) A working model illustrating that S100A8-mediated NLRP3 inflammasome-dependent pyroptosis in macrophages facilitates liver fibrosis progression, and that the identified GSDMD may be used as a potential biomarker during liver fibrosis onset and progression.

Based on the role of S100A8-elicited NLRP3 pyroptosis in liver fibrosis, we chose a well-defined cohort of liver fibrosis patients to assess the clinical importance of circulating S100A8, GSDMD, IL-1β, and IL-18 for predicting the occurrence and progression of disease. The ROC analysis indicated that the circulating GSDMD had the strongest diagnostic value for the occurrence of liver fibrosis with an area under the ROC curve (AUC) of 0.95 (95% CI, 0.9279–0.9842) compared to S100A8, IL-1β or IL-18 with AUCs of 0.93 (95% CI, 0.9011–0.9766), 0.81 (95% CI, 07523–0.8849), and 0.81 (95% CI, 0.7425–0.8803), respectively (Figure 6A). Moreover, we explored the predictive ability of these indicators for liver fibrosis severity. Furthermore, circulating GSDMD had the highest diagnostic value for identifying severe liver fibrosis, which yielded an AUC of 0.91 (95% CI, 0.8614–0.9725) compared to IL-1β, S100A8, and IL-18 with AUCs of 0.90 (95% CI, 0.8523–0.9677), 0.89 (95% CI, 0.8209–0.9606), and 0.89 (95% CI, 0.8348–0.9591), respectively (Figure 6B). These data implied that the identified GSDMD may be used as a potential biomarker during the occurrence and progression of liver fibrosis.

## 4. Discussion

NLRP3 inflammasome-dependent pyroptosis, an identified inflammatory form of cell death, is activated by two signals; namely, priming and activating signals, leading to persistent inflammation via activation and release of IL-1β, IL-18, and other intracellular contents [7]. Recently, pyroptosis has attracted interest due to its crucial role in inflammation-related diseases [31]. Intrahepatic cell death and persistent inflammation triggered by various etiological factors are two central elements in the occurrence and progression of liver fibrosis. Evidence supports that NLRP3 inflammasome-dependent pyroptosis is involved in the development of liver fibrosis [32]. Nevertheless, the definite cell types that undergo pyroptosis and the underlying mechanism as well as their clinical importance are still unclear. In the present study, we demonstrated that S100A8 as a crucial DAMP could stimulate NLRP3 inflammasome-dependent pyroptotic macrophage death by activating TLR4-dependent NF-κB and inducing ROS abundance, finally facilitating liver fibrosis progression. In addition, we identified that the pyroptosis-related indicator GSDMD may be a potential biomarker for the occurrence and progression of liver fibrosis (Figure 6C).

The role of pyroptosis has been extensively confirmed in a wide range of fibrotic responses ranging from the lung, kidney, heart, and skin. In these fibrotic diseases, the interaction between the NF-κB/NLRP3/caspase-1/IL-1β axis and TGF-β signaling appears to be the main mechanism relevant to fibrosis [8,9,10,33,34]. Here, elevated levels of the pyroptosis-related indicators GSDMD, IL-1β, and IL-18 were confirmed in both clinical specimens from liver fibrosis patients and CCl4-induced liver fibrosis mouse models. Additionally, their serum levels strongly correlated with the severity of liver fibrosis, suggesting the essential role of pyroptosis during the progression of liver fibrosis. Furthermore, double immunofluorescence staining of NLRP3 with albumin, F4/80 or with α-SMA in human and mouse fibrotic liver tissues demonstrated that enhanced NLRP3 was mainly localized to KCs and hepatocytes, especially KCs, indicating that KCs are major cell types that undergo pyroptosis, which is consistent with other studies regarding these cell types [35]. Targeting the NLRP3 inflammasome and its downstream effectors may be a potent therapeutic strategy for inflammatory diseases [36]. Here, we investigated whether NLRP3 inflammasome-dependent pyroptosis could be an intervention target for liver fibrosis. We used a specific inhibitor of NLRP3, MCC950, to treat CCl4-induced liver fibrosis mouse models. As expected, injection of MCC950 significantly attenuated liver injury, especially liver fibrosis and improved liver function, indicating that NLRP3, as the executor of pyroptosis, is an advancing prevention target for liver fibrosis. Additionally, the effectiveness of the NLRP3 inhibitor was confirmed by the CM (pyroptotic macrophages)-LX-2 culture model. In previous studies, inhibitors of IL-1 signaling and caspase-1 were also effective in treating NLRP3-driven diseases [36]. Therefore, further studies are still needed to compare the effects of these inhibitors with MCC950 to screen out the optimal inhibitors for liver fibrosis.

DAMPs refer to many endogenous molecules with immunomodulatory activity released from stressed, malfunctioning or dead cells and damaged tissues [37]. It has been reported that DAMPs released from dying tubule cells, including HGMB1, contribute to the macrophage infiltration and IL-1β release, which markedly facilitates renal fibrogenesis [25]. Another study also suggested the involvement of citrullinated vimentin derived from lung macrophages as a DAMP during the progression of lung fibrosis [26]. As two members of DAMP, S100A8 and S100A9, were reported to correlate with the onset and progression of bone marrow fibrosis [38] and renal fibrosis [39]. Similarly, elevated levels of S100A8 and S100A9 were verified in clinical samples and CCl4-induced mouse model studies, and their levels were strongly related to the severity of liver fibrosis. Specifically, S100A8 increased more dramatically than S100A9 during the progression of liver fibrosis, implying an important role of S100A8 in the pathogenesis of liver fibrosis. The present data, together with the above-mentioned results from other studies, further emphasize the significance of DAMPs in fibrotic disease. S100A8 and S100A9 are mainly derived from activated immunocytes (neutrophils, macrophages, etc.) and cells in local lesions in many inflammatory processes [40]. Studies have shown that numerous pro-inflammatory cytokines, including tumor necrosis factor-a (TNF-a) and interleukin-1 (IL-1), strongly induce the expression of S100A8/A9 [41]. Moreover, LPS activates caspase-4/5 inflammasome and promotes the secretion of S100A8 from macrophages. The proximal promoter regions of S100A8 and S100A9 have common binding sites for different transcription factors (e.g., AP-1, NF-κB, and C/EBP) [41]. However, the detailed molecular mechanism controlling the transcription of S100A8/A9 genes during liver fibrosis process is necessary for further extended analysis in future studies.

Recently, S100A8 and S100A9 were reported to activate NLRP3 inflammasome signaling to promote the pathogenesis of several diseases [29,42]. Given that the NLRP3 inflammasome can respond to DAMPs as a classical PRR, we focused on whether S100A8 could activate the NLRP3 inflammasome and subsequently lead to pyroptosis. In the present study, we observed a close correlation between S100A8 and pyroptosis, and found a direct effect of S100A8 on macrophage pyroptosis. Additionally, pyroptotic products were able to induce the activation of HSCs. The augmenting inflammatory factor IL-1β was detected in CM, which is a critical profibrotic cytokine that acts on HSCs in previous reports [43]. In addition to IL-1β, other profibrotic cytokines released from damaged cells, such as HGMB1, ATP, and DNA, can trigger HSC activation and collagen production [44], which needs further study for confirmation. Activation of NLRP3 inflammasome-dependent pyroptosis requires two signals, the priming signal and the activating signal. With regard to the priming signal, our data showed that S100A8 interacted with TLR4 and then activated downstream NF-κB with transcriptional upregulation of NLRP3, pro-IL-1β, and pro-IL-18. ROS have been proposed as the second signal for NLRP3 activation [45], and they also appear to play a crucial role in fibrotic progression [46]. In addition, S100A8 was reported to regulate ROS production by increasing NADPH oxidase activity [29,30]. Therefore, we focused on the ROS-mediated second activation signal. In this study, S100A8 significantly enhanced ROS levels in THP-1 macrophages. Furthermore, suppressing ROS generation with the NOX specific inhibitor DPI markedly attenuated S100A8-induced NLRP3 activation. Interestingly, the use of DPI also decreased the levels of the priming signaling molecules pro-IL-1β and total GSDMD mediated by S100A8, suggesting that S100A8-induced ROS production may exhibit a crosstalk with the priming signal NF-κB activation, which was supported by other studies [29,47]. A previous study also suggested that ROS can be generated from mitochondria in a TLR4-dependent manner [48]. Here, it is still unclear whether S100A8-induced ROS production is dependent on TLR4, which requires confirmation in further studies. Collectively, we demonstrated that S100A8 not only induced transcriptional upregulation of NLRP3, pro-IL-1β, and pro-IL-18 via TLR4/NF-κB signaling, but also facilitated oligomerization of NLRP3 proteins and cleavage of caspase-1 through NOX/ROS signaling, finally leading to pyroptotic cell death in macrophages. Recently, we discovered that CD36, which is expressed on the surface of a variety of cells, including macrophages, hepatocytes, enterocytes, myocytes, and adipocytes, also acts as a receptor of S100 family proteins (S100A8, S100A9, and S100A12) [49]. CD36 is involved in many pathophysiological processes, such as cardiovascular, thrombotic, and metabolic phenotypes [50]. However, there are few reports on its role in liver fibrosis. It has been shown that in the presence of DAMPs, CD36 assembles and interacts with other membrane receptors, leading to ROS production and transcription factor activation [51]. Therefore, we wondered whether CD36 mediates S100A8-induced ROS production and NF-κB pathway activation in liver fibrosis progression, which requires further studies to investigate.

Currently, the diagnosis of liver fibrosis mainly depends on liver biopsy supplemented with serology tests and imaging examinations [52]. However, liver biopsy is an invasive method with potential associated complications and mortality [53], and conventional ultrasonography, CT and MRI have little diagnostic significance for early-stage liver fibrosis [54]. Due to their high applicability, good interlaboratory reproducibility, and potential widespread use, serum biomarkers are still the optimal option for liver fibrosis examination. Although there are some identified serum biomarkers, none are liver-specific [54]. Therefore, it is still necessary to identify several new promising serum biomarkers for the diagnosis and staging of liver fibrosis in combination with known good indicators to improve diagnostic power. In this study, since S100A8-induced NLRP3 inflammasome-dependent pyroptosis is correlated with liver fibrosis, its diagnostic value for the onset and progression of liver fibrosis was analyzed. The identified circulating GSDMD had the highest diagnostic value for the diagnosis and staging of liver fibrosis, suggesting that GSDMD may have the potential to be an alternative biomarker for liver fibrosis evaluation. Nevertheless, there are still limitations in our study. Due to the small sample size and lack of specific investigation of liver fibrosis with different etiologies, further research is required in more liver fibrosis patients with different etiologies to confirm these data.

In conclusion, the current study suggests that S100A8 stimulates NLRP3 inflammasome-dependent pyroptosis in macrophages via activating TLR4/NF-κB signaling and inducing ROS abundance, which finally facilitates the progression of liver fibrosis. In addition, the NLRP3 inhibitor MCC950 treatment reduced the development of liver fibrosis in CCl4-induced liver fibrosis mouse models, indicating that blocking NLRP3 inflammasome-dependent pyroptosis may be a promising therapeutic strategy. More importantly, the identified pyroptosis-related indicator GSDMD has the potential to be an alternative biomarker for liver fibrosis evaluation.

## Figures and Tables

**Table 1:** The characteristics of enrolled individuals.

Parameters	Liver fibrosis	HCs	
	Serum/tissue Specimen (*n* = 89)	Serum specimen (*n* = 60)	Tissue Specimen (*n* = 5)
**Gender**			
Male *n* (%)	47 (52.81)	34 (56.66)	3 (60)
Fale *n* (%)	42 (47.19)	26 (43.33)	2 (40)
**Age (years) (IQR)**	57 (12.75)	57 (14.5)	59 (17.5)
**Aetiology**			
Viral hepatitis *n* (%)	42 (47.19)	NA	NA
Cholestatic /Autoimmune *n* (%)	25 (28.08)	NA	NA
Alcohol *n* (%)	16 (17.97)	NA	NA
Others *n* (%)	6 (6.74)	NA	NA
**Stage of fibrosis (F)**			
F0 *n* (%)	9 (10.11)	NA	NA
F1 *n* (%)	10 (11.23)	NA	NA
F2 *n* (%)	20 (22.47)	NA	NA
F3 *n* (%)	29 (32.58)	NA	NA
F4 *n* (%)	21 (23.59)	NA	NA

Abbreviations: IQR, interquartile range; HCs, healthy controls; NA, not applicable.

**Table 2:** Sequence of primers used for quantitative RT-PCR.

Genes	Forward (5′-3′)	Reverse (5′-3′)
*NLRP3*	CTTCTCTGATGAGGCCCAAG	GCAGCAAACTGGAAAGGAAG
*pro-IL-1β*	TCCAGGGACAGGATATGGAG	TCTTTCAACACGCAGGACAG
*pro-IL-18*	AAGATGGCTGCTGAACCAGT	GAGGCCGATTTCCTTGGTCA
*Col1a1*	AAGAGTGGAGAGTACTGGATT	GTTCTTGCTGATGTACCAGT
*α-SMA*	CGTGGGTGACGAAGCACAG	GGTGGGATGCTCTTCAGGG
*TGF-β*	GGCCAGATCCTGTCCAAGC	GTGGGTTTCCACCATTAGCAC
*GAPDH*	CCACTCCTCCACCTTTGAC	ACCCTGTTGCTGTAGCCA

Abbreviations: NLRP3, nod-like receptor protein-3; COL1A1, collagen I; α-SMA, α-smooth muscle actin; TGF-β, transforming growth factor beta; GAPDH, glyceraldehyde-3-phosphate dehydrogenase.

## Data Availability

The datasets used and/or analyzed during the current study are available from the corresponding author on reasonable request.

## Supplementary Materials

The following supporting information can be downloaded at: https://www.mdpi.com/article/10.3390/cells11223579/s1, Figure S1: The mean of IOD analysis for all IHC staining utilizing the Image Pro Plus 6.0 (MediaCybernetics, Silver Spring, MD, USA). Figure S2: ELISA analysis for IL-1β levels in supernatants from THP-1 treated with 5 µg/mL GST or rhS100A8.
