# Supplementary material for: S100A8-Mediated NLRP3 Inflammasome-Dependent Pyroptosis in Macrophages Facilitates Liver Fibrosis Progression
Source: Cells. 2022 Nov 12;11(22):3579. doi: 10.3390/cells11223579 (PMC9688473; doi:10.3390/cells11223579)
Supplement: Supplementary file 1 [file cells-11-03579-s001.zip › cells-2008899-supplementary.pdf]

cells

MDPI

Supplementary Materials:

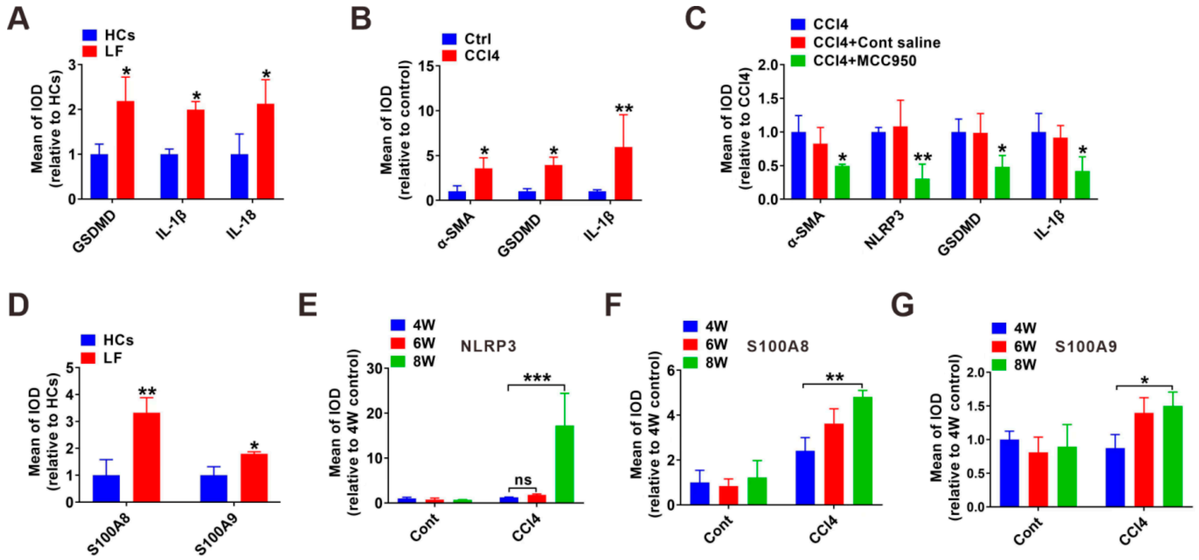

Figure S1. The mean of integrated optical density (IOD) analysis for all IHC staining utilizing the Image Pro Plus software. (**A**) The levels of GSDMD, IL-1β and IL-18 in liver sections from liver fibrosis patients were quantified by densitometry and are shown as fold changes relative to the HCs. (**B**) The levels of α-SMA, GSDMD and IL-1β in liver sections from CCl4 group mouse were quantified by densitometry and are shown as fold changes relative to vehicle group mouse. (**C**) The levels of α-SMA, NLRP3, GSDMD and IL-1β in liver sections from CCl4+Cont saline group mouse or CCl4+MCC950 group mouse were quantified by densitometry and are shown as fold changes relative to CCl4 group mouse. (**D**) The levels of S100A8 and S100A9 in liver sections from liver fibrosis patients were quantified by densitometry and are shown as fold changes relative to the HCs. (**E**–**G**) The levels of NLRP3 (**E**), S100A8 (**F**) and S100A9 (**G**) in liver sections from CCl4 group mouse were quantified by densitometry and are shown as fold changes relative to vehicle group mouse. ns, not significant; * $p < 0.05$, ** $p < 0.01$, *** $p < 0.001$.

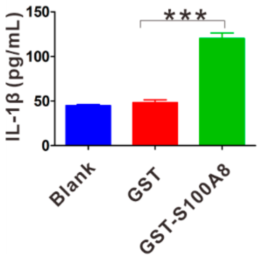

Figure S2. ELISA analysis for IL-1β levels in supernatants from THP-1 treated with 5 μg/ml GST or rhS100A8. *** $p < 0.001$.
